# Combined effects of normobaric hypoxia and cold on respiratory system responses to high‐intensity exercise

**DOI:** 10.1113/EP092468

**Published:** 2025-05-11

**Authors:** Alexa Callovini, Alessandro Fornasiero, Aldo Savoldelli, Gianluigi Dorelli, Marco Decet, Lorenzo Bortolan, Barbara Pellegrini, Federico Schena

**Affiliations:** ^1^ CeRiSM, Sport Mountain and Health Research Centre University of Verona Rovereto Italy; ^2^ Department of Engineering for Innovation Medicine University of Verona Verona Italy; ^3^ Department of Cellular, Computational and Integrative Biology University of Trento Trento Italy; ^4^ Department of Neurosciences, Biomedicine and Movement Sciences University of Verona Verona Italy

**Keywords:** cold exposure, exercise‐induced bronchoconstriction, high‐intensity exercise, normobaric hypoxia, respiratory responses

## Abstract

Cold temperatures (<−15°C) increase exercise‐induced bronchoconstriction (EIB), while hypoxic‐induced hyperventilation exacerbates respiratory muscle fatigue for a given exercising task. This study aimed to determine the individual and combined effects of cold and normobaric hypoxia on the respiratory system responses to high‐intensity exercise. Fourteen trained male runners (${\dot{V}}_{O_{2}\max}$: 64 ± 5 mL/kg/min) randomly performed an incremental cardiopulmonary exercise test (CPET) to volitional exhaustion under four environmental conditions: normothermic (18°C) normoxia ($F_{IO_{2}}$: 20.9%) and hypoxia ($F_{IO_{2}}$: 13.5%), and cold (−20°C) normoxia and hypoxia. Ventilatory responses during exercise and lung function (LF), maximal inspiratory (MIP) and expiratory (MEP) pressure measurements before and after exercise were evaluated. Volume of air forcefully exhaled in 1 s (FEV1), FEV1/forced vital capacity (FVC), peak expiratory flow, forced expiratory flow during the mid (25–75%) portion of the FVC, and maximal expiratory flow at 50% of FVC were affected by cold exposure. No significant pre‐ to post‐exercise change in MIP and MEP was found, independent of environmental conditions. Greater LF impairments in cold‐normoxia and coldhypoxia were associated with the lowest peak ventilatory responses during exercise. Cold exposure was found to negatively impact peak ventilatory responses and post‐exercise LF, further highlighting a relationship between EIB presence and the blunted ventilatory response in the cold. Respiratory muscle strength remained unchanged after exercise regardless of the environmental condition, suggesting no detrimental effect of hypoxia on this parameter when intermittent short‐duration high‐intensity exercises are performed. Future studies should investigate the combined cold‐hypoxic effect on longer exercise durations at a sustained high intensity, accounting for differences between normobaric and hypobaric hypoxia exposures.

## Abstract (highlights)

- **What is the central question of this study?**
  What are the independent and combined effects of cold and normobaric hypoxia on respiratory responses to high‐intensity exercise?
- **What is the main finding and its importance?**
  Cold exposure impaired lung function and peak ventilatory responses during high‐intensity exercise, with greater impairments observed under combined cold‐hypoxia condition. The findings highlight a link between exercise‐induced bronchoconstriction and reduced ventilatory capacity in cold environments. Respiratory muscle strength remained unaffected post‐exercise across all conditions, suggesting no detrimental impact of hypoxia during short‐duration high‐intensity tasks.

## 1 INTRODUCTION

During exercise, minute ventilation (${\dot{V}}_{E}$) must increase to ensure sufficient oxygen delivery and carbon dioxide elimination to match the elevated metabolic demands of active tissues (Peters et al., 2023). This rise in ${\dot{V}}_{E}$ is mediated by coordinated adjustments in tidal volume (*V*_t_) and respiratory frequency (*f*_R_). More specifically, up to moderate intensity exercise, a ventilatory strategy favouring an increase in *V*_t_ enables adequate alveolar ventilation while preventing excessive intra‐abdominal pressure (Gibson et al., 2002). However, during heavier exercise, further increases in *V*_t_ become too costly with respect to the work of breathing (WOB), and increases in *f*_R_ are primarily responsible for further increases in ${\dot{V}}_{E}$. This finely tuned regulation allows the diaphragm to operate near its optimal length for force generation, minimizing the WOB and delaying the onset of respiratory muscle fatigue (RMF), defined as a temporary loss in force or speed capacity that resolves with rest (Oueslati et al., 2018). Furthermore, maintaining or even increasing airway calibre during exercise is essential to prevent increased WOB; in fact, the relative O_2_ cost of respiratory muscles at maximal exercise accounts for ∼5–10% of ${\dot{V}}_{O_{2}\max}$ in healthy subjects (Aaron et al., 1992), but it can rise to 15% in the presence of expiratory flow limitations, possibly becoming a limiting factor of maximal exercise performance in some subjects (Vella et al., 2006).

Despite these adaptations aimed at sustaining diaphragmatic force for effective ventilation, increased respiratory muscle work and early fatigue onset occur when exercising in hypoxia (Dempsey et al., 2006; Vergès et al., 2005). In fact, a reduced partial pressure of oxygen in arterial blood ($P_{aO_{2}}$) acts as a primary signal for rapidly heightened ventilation (Ainslie et al., 2013; Calbet & Lundby, 2009). As a result, ${\dot{V}}_{E}$ becomes disproportionately elevated for a given absolute exercise intensity in acute hypoxia compared to normoxia, increasing WOB and reducing exercise capacity in this context (Price, 2014). Notably, even when the ventilatory load on respiratory muscles in hypoxia is similar to that in normoxia (i.e. similar ventilation with a reduced workload in hypoxia), early fatigue can still arise due to reduced oxygen delivery to these muscles (Vogiatzis et al., 2007).

Apart from reduced oxygen availability, high‐altitude exposure often involves freezing air temperatures, which are a known trigger of exercise‐induced bronchoconstriction (EIB). EIB is characterized by the narrowing of intrathoracic airways resulting from smooth muscle constriction triggered by airway inflammation following exercise‐induced hyperventilation (Bonini, 2018; Parsons et al., 2013). Since airflow obstruction and related symptoms typically arise after exercise has ended, it is generally believed that EIB does not affect airflow during exercise itself and, as such, may not significantly impact exercise performance (Gotshall, 2006; Stirling et al., 1983). However, recent studies (Mediano et al., 2017; Price, 2014) show that individuals with EIB adopt altered ventilatory strategies during exercise to counteract airflow limitations occurring already while exercising.

Thus, environmental factors such as cold and hypoxia compound the physiological stress placed on the respiratory system, potentially affecting exercise responses and capacity. While the separate effects of cold (Carey, 2015; Kennedy & Faulhaber, 2018; Kennedy et al., 2020; Sandsund et al., 1997) and hypoxia (Babcock et al., 1995; Gudjonsdottir et al., 2001; Vergès et al., 2005; Vogiatzis et al., 2007) on whole‐body exercise respiratory responses have been widely researched, the combined effects of these two stressors remain unclear (Hinde et al., 2018). Interestingly, our research group (Callovini et al., 2023) recently demonstrated that workload (WL_max_) and tidal volume (*V*_tmax_) exhibited a complete additive reduction effect of cold and hypoxia following a maximal cardiopulmonary exercise test to exhaustion, meaning that the decrease of above‐mentioned variables by the combination of cold and hypoxia was equal to the sum of the effects exerted by the two environmental stressors alone (Lloyd & Havenith, 2016). However, ${\dot{V}}_{\mathrm{Emax}}$ showed only a partial additive reduction effect in the cold‐hypoxic environment (i.e. the decrease in ${\dot{V}}_{E}$ in the cold‐hypoxic condition was smaller than the sum of the individual effect of cold and hypoxia on this parameter), underscoring the need to better understand the mechanisms underlying the ventilatory strategy adopted in this setting and its potential impact on maximal exercise capacity under combined stressor exposures.

For these reasons, the primary aim of this study was to examine respiratory muscle strength (as a marker of fatigue) and lung function responses following maximal exercise to exhaustion in healthy subjects, considering both the independent and combined effects of hypoxia and cold exposure on these aspects. Moreover, correlations between variations in these responses and the ventilatory pattern adopted at maximal exercising intensities is investigated. We hypothesize that cold‐induced EIB and/or hypoxia‐induced RMF are related to modifications in ventilatory responses to maximal exercise under single and combined stressor exposure, one of the possible mechanisms behind further reduced exercise capacity being the simultaneous presence of these phenomena (i.e. additive effect of cold and hypoxia on WL_max_) in this latter condition (Callovini et al., 2023). This research will help in gaining insights into the physiological adaptations and health risks associated with exercise in extreme environments.

## 2 METHODS

### 2.1 Ethical approval

This study was reviewed and approved by the local ethics committee (University of Verona – Project No. 4105CESC) and conformed to the *Declaration of Helsinki*. Before data collection, all participants were adequately informed about the experimental procedures and gave their written informed consent for the measurements.

### 2.2 Subjects

Fourteen trained (De Pauw et al., 2013) male subjects volunteered for this study (Table 1). All participants were non‐smokers, free of any systemic or chronic illness, and not taking medications. They all had a valid sports medical examination, with no contraindications reported regarding cardiac or respiratory aspects. Baseline spirometry values were within normal ranges, and none of the subjects claimed to have ever experienced adverse respiratory symptoms due to high‐intensity exercise. None of the participants had a physician‐confirmed diagnosis of asthma, and exclusion criteria encompassed a predisposition to atopic conditions. Thirteen subjects completed all experimental sessions, whereas one subject completed four out of five sessions.

**TABLE 1:** Subjects characteristics and baseline pulmonary function evaluation.

Characteristic	Value
Age (years)	27.2 ± 3.4
Height (cm)	177.2 ± 4.5
Weight (kg)	70.2 ± 5.3
BMI (kg/m^2^)	22.4 ± 1.7
${\dot{V}}_{O_{2}\max}$ (mL/kg/min)	64.0 ± 5.2
HR_max_ (bpm)	191 ± 6
FVC (L)	5.55 ± 0.59
FVC (% predicted)	101.9 ± 9.9
FEV1 (L)	4.39 ± 0.50
FEV1 (% predicted)	97.0 ± 10.2
FEV1/FVC (ratio)	79.12 ± 4.97
FEV1/FVC (% predicted)	94.6 ± 5.2
FEF_25–75%_ (L/s)	3.98 ± 0.94
FEF_25–75%_ (% predicted)	85.0 ± 19.1

Data are reported as means ± SD, (overall *n* = 14). Abbreviations: BMI, body mass index; HR_max_, maximal heart rate; FEV1, forced expiratory volume in 1 s; FVC, forced vital capacity; FEF_25–75%_, forced expiratory flow at 25–75%.

### 2.3 Preliminary assessment and experimental design

Each participant underwent five laboratory visits, including an initial assessment and four subsequent experimental trials, all scheduled at the same time of the day.

During the preliminary session, a baseline spirometry assessment was completed prior to an incremental test to exhaustion on a motorized treadmill (slope: 25%, starting speed 2.0 km/h increased by 0.7 km/h every 3 min), through which subjects’ ${\dot{V}}_{O_{2}\max}$ and individual maximal ascensional velocity were determined. Cardiorespiratory measures were collected continuously with a breath‐by‐breath method using an automated open‐circuit gas analysis system (Quark PFT Ergo, Cosmed Srl, Rome, Italy), and heart rate (HR) was recorded continuously during the test. The results were used to define individual running speed in the exercise protocols for the four experimental trials.

All four sessions following the preliminary assessment were performed in an environmental chamber where it was possible to vary the ambient temperature and simulate high‐altitude exposure (i.e. normobaric hypoxia). The hypoxic environment was created through the manipulation of the $F_{IO_{2}}$ by means of an oxygen dilution system based on the vacuum pressure swing adsorption principle (B‐Cat, Tiel, The Netherlands). $F_{IO_{2}}$ was set at 13.5% to simulate an altitude of 3500 m a.s.l. The temperature was regulated using a particular air conditioning and refrigeration system (Frigotherm Ferrari SRL, Lana, Italy). Relative humidity was set at 40% in all conditions.

During these sessions, participants were randomly exposed to each of the following conditions: normothermic normoxia (N: 18°C, 20.9% $F_{IO_{2}}$), normothermic hypoxia (H; 18°C, 13.5% $F_{IO_{2}}$), cold normoxia (C: −20°C, 20.9% $F_{IO_{2}}$) and cold hypoxia (CH: −20°C, 13.5% $F_{IO_{2}}$). They were blind to the $F_{IO_{2}}$ value but not to temperature conditions.

There was a minimum of 48 h between each experimental session. All participants completed the protocol within a 6‐week period between March and November so that no cold‐acclimatization was present. Subjects were allowed to continue their normal exercise and activity patterns. However, they were asked to refrain from intense physical activity on the day before and from drinking any alcohol and caffeinated beverages on the day of the test.

### 2.4 Experimental trials

The main test sessions started with pre‐trial spirometry and respiratory muscle pressure measurements outside of the chamber at a normal indoor ambient temperature of 20°C. Participants were then equipped with a heart rate chest strap (Polar, Kempele, Finland) and invited to wear appropriate clothing depending on the environmental temperature of that specific session. During cold conditions, participants wore individually chosen extreme cold weather technical clothing (including winter sports jacket/sweater, trousers, gloves, and hat or band (estimated clothing insulation in the cold: 1.50 clo), which remained identical for C and CH trials; however, they were not allowed to cover the face or mouth in any manner (scarf, buff, hand) throughout the whole exercise session, but could dress or undress as they felt comfortable during both rest and exercise. To ensure the occurrence of first short‐term physiological responses to the hypoxic environment, once subjects entered the chamber, they remained seated for a 30‐min resting period, already exposed to the specific environmental condition (Duffin, 2007). This was repeated within all conditions to guarantee participants’ blindness to ambient $F_{IO_{2}}$. During cold trials, additional blankets were provided for the 30‐min resting period to prevent excessive cooling of the core and extremities.

The exercise protocol started with a 10‐min warm‐up phase (2 km/h, slope 25%) followed by a submaximal to maximal test of 4‐min intervals at increasing velocities (cardiopulmonary exercise test, CPET), interspersed by 2 min of passive recovery performed in standing position on the treadmill using handrail support. Treadmill inclination was kept constant at 25% (Fornasiero et al., 2020), whereas the test's speed started from 30% of the individual maximal speed measured at the pretest and increased by 10% every interval until exhaustion. The test ended when the participant was unable to complete 4 min of exercise at the prescribed load. Respiratory muscle pressure measurements were repeated inside the environmental chamber 2 min after volitional exercise cessation. Immediately after, subjects were asked to re‐start exercising on the treadmill and reach within 4 min the speed of the last completed step of the previously performed CPET (∼90% of specific condition maximal workload), then complete four additional minutes at that speed or, if not possible due to insurgence of fatigue, at the maximal sustainable speed. An exercising intensity equal to at least 85% of maximal HR and 80% of maximal ${\dot{V}}_{E}$ measured at the end of the CPET was always reached (Weiler et al., 2016).

**FIGURE 1:**
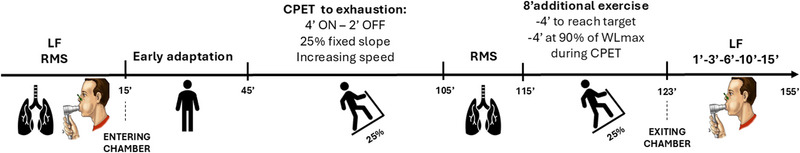
Schematic representation of the study design. CPET, cardiopulmonary exercise test; LF, lung function; RMS, respiratory muscles strength; WL_max_, maximal workload.

This last exercising bout was projected to investigate EIB insurgence adequately by performing spirometry at 1, 3, 6, 10 and 15 min post‐exercise (1‐Post; 3‐Post; 6‐Post; 10‐Post; 15‐Post) outside of the environmental chamber, in accordance with previous methods (Kennedy & Faulhaber, 2018; Kennedy et al., 2020). During this phase, participants were allowed to walk around slowly to provide a typical cool down found after exercise. A schematic representation of the study design is presented in Figure 1.

Due to the extremely cold conditions, collecting cardiorespiratory measures continuously through the automated open‐circuit gas analysis system was impossible. However, at rest and during the last 40 seconds of each exercise intensity (when a steady state of ${\dot{V}}_{O_{2}}$ was assumed to be reached), ventilatory data were collected using a flowmeter connected to a measuring system purpose‐built for this project by our engineers. The flowmeter used was that of the Quark PFT system and it was calibrated with a 3‐L syringe following exactly the instructions of the open‐circuit gas analysis system.

### 2.5 Lung function and respiratory muscle strength

Lung function tests were performed using an ergospirometer (Quark PFT, Cosmed, Rome, Italy) in accordance with the guidelines of the European Respiratory Society (ERS) (Graham et al., 2019) and by trained personnel to ensure consistency of the procedure.

The main outcome measures were forced expiratory volume in 1 s (FEV1), forced vital capacity (FVC), the ratio of FEV1 to FVC (FEV1/FVC%), maximal expiratory flow at 50% of FVC (MEF_50%_), the average forced expiratory flow during the mid (25–75%) portion of the FVC (FEF_25–75%_) and peak expiratory flow (PEF) (Kennedy & Faulhaber, 2018; Kennedy et al., 2020, 2019; Sandsund et al., 1997). All manoeuvres complied with the general acceptability criteria of the ERS.

Also maximal voluntary inspiratory (MIP) and expiratory (MEP) pressure were measured before exercise and within 4 min of CPET completion in order to evaluate exercise‐induced changes in respiratory muscle strength (RMS) as an indication of respiratory muscle fatigue (Oueslati et al., 2018). Participants were asked to produce maximal inspiration or expiration through a mouthpiece into an occluded non‐deformable tube (Gibson et al., 2002). A small leak (1 mm diameter) was used to prevent glottis closure. The tube was attached to a negative and positive pressure gauge depending on the performed manoeuvre (MIP or MEP, respectively). Measurement of MIP was initiated at maximal expiratory lung volume and MEP at maximal inspiratory lung volume and lasted a minimum of 3 s. Maximal efforts were repeated at least three times with a minimum of 30 s between measures until there were at least two maximal values within 10% variance (McConnell et al., 1997), and the highest value was subsequently used for analysis (Hinde et al., 2020). Considering the high variability in performing these tests within the same subject, each participant had a comprehensive familiarization with the manoeuvres and received verbal encouragement to maintain a maximal effort throughout all sessions. All manoeuvres were performed standing with subjects’ backs leaning on the wall to avoid abdominal muscle contraction during trials.

### 2.6 Data analysis

Ventilatory data were processed and analysed with MATLAB 7.0 (The MathWorks, Inc., Natick, MA, USA), using a customized code. ${\dot{V}}_{E}$, *f*_R_ and *V*_t_ at maximal exercise were averaged over the last 40 s registered during the last or the last but one stage of the CPET (since in some cases subjects completed less than 2 min during the last stage and given the 2‐min recovery phase between stages, at the end of the test some parameters were still rising), and during the last minute of exercise before lung function (LF) evaluation.

Absolute maximum Pre‐to‐Post change in spirometry values (FVC, FEV1, FEV1/FVC, PEF, MEF_50%_, and FEF_25–75%_) were calculated in raw units as well as maximum percentage change ((pre‐exercise − minimum post‐exercise)/(pre‐exercise value) × 100) based on a previously published protocol (Stensrud et al., 2007). The minimum post‐exercise value was selected considering the time point after exercise which presented the maximum delta from pre‐trial values (i.e. each trial may present the lowest post‐exercise value at a different time point). Moreover, the absolute changes in spirometry measures from all post‐trial time points minus the pre‐trial value were calculated, and then relative changes were derived as explained above (Kennedy et al., 2020). Absolute and relative changes from pre to selected post‐exercise MIP and MEP were also calculated (Hinde et al., 2018).

### 2.7 Statistical analysis

Descriptive analysis was used to report the results (the mean ± SD). All the data were tested for their normal distribution (Shapiro–Wilk test). In the first step, possible differences in spirometry and MIP and MEP pre‐trial values before being exposed to the environmental condition between experimental sessions were tested using a one‐way repeated‐measures ANOVA with ‘condition’ (1 vs. 2 vs. 3 vs. 4) as a within‐subjects factor.

Generalized estimating equation (GEE) analysis was used to test the main effects of ‘$F_{IO_{2}}$ ’ (20.9% vs. 13.5%) and ‘temperature’ (+18°C vs. −20°C), as well as their ‘interaction’, on maximum relative changes (using minimum post‐exercise value, independently of considered time point) in spirometry (FVC, FEV1, FEV1/FVC, PEF, MEF_50%_ and FEF_25–75%_) and respiratory muscle strength (MIP and MEP) parameters. When an interaction effect ($F_{IO_{2}}$ × temperature) was found, Šidák's *post hoc* test was used for specific comparisons (Cunha et al., 2015). Subsequently, relative changes for spirometry outcomes at each post‐exercise time point were analysed using GEE with ‘$F_{IO_{2}}$ ’, ‘temperature’ and ‘time’ (1‐Post, 3‐Post, 6‐Post, 10‐Post and 15‐Post) as within‐subjects factors. When an interaction effect (‘$F_{IO_{2}}$ × temperature’, ‘$F_{IO_{2}}$ × time’, ‘temperature × time’ or ‘$F_{IO_{2}}$ × temperature × time) was found, Šidák's *post hoc* test was used for specific comparisons. GEE was implemented to analyse data since one subject did not complete all experimental sessions, and another presented some missing data in post‐exercise measurements in one of the experimental sessions. All data were analysed using a standard statistical package (SPSS Statistics, IBM Corp., Armonk, NY, USA).

Finally, repeated measures correlations (https://lmarusich.shinyapps.io/shiny_rmcorr/) were used for determining the common within‐individual association between maximal (${\dot{V}}_{\mathrm{Emax}}$, *V*_tmax_ and *f*_Rmax_) ventilatory responses and maximum relative changes in lung function (FVC, FEV1, FEV1/FVC, PEF, MEF_50%_ and FEF_25–75%_) and MIP and MEP parameters.

The threshold for statistical significance was set at *P <* 0.05.

## 3 RESULTS

### 3.1 Exercising ventilatory parameters and maximal workload

Main decreasing effects of hypoxia (all *P <* 0.05) and cold (all *P <* 0.05) without interaction were found for ${\dot{V}}_{\mathrm{Emax}}$ (N: 163.3 ± 21; H: 152 ± 20.6; C: 139.5 ± 20.7; CH: 136.2 ± 25.6 L/min) and *V*_tmax_ (N: 2.82 ± 0.4; H: 2.6 ± 0.5; C: 2.6 ± 0.6; CH: 2.3 ± 0.5 L/min). Oppositely, no general hypoxic or cold effect, but a ‘temp × $F_{IO_{2}}$ ’ interaction effect (*P =* 0.013) was seen for *f*_Rmax_ (N: 64 ± 11; H: 62 ± 11; C: 61 ± 11; CH: 65 ± 13 bpm), which was significantly higher in CH than in C and H alone. Main effects of hypoxia (*P <* 0.001) and cold (*P <* 0.001) were found for WL_max_, which was lower in H (5.5 ± 0.5 km/h) and C (6.6 ± 0.6 km/h) compared to N (6.8 ± 0.6 km/h), with no further significant reduction in CH (5.3 ± 0.5 km/h) (Callovini et al., 2023).

### 3.2 Lung function and respiratory muscle strength

#### 3.2.1 Pre‐trial values (Supporting information, Table S1)

No differences in Pre values between conditions were found for MIP (*P =* 0.577) and MEP (*P =* 0.980). Similarly, no differences in Pre FVC (*P =* 0.944), FEV1 (*P =* 0.859), FEV1/FVC% (*P =* 0.954), PEF (*P =* 0.893), FEF_25–75%_ (*P =* 0.900) and MEF_50%_ (*P =* 0.939) were detected between N, H, C and CH conditions.

### 3.3 Maximum relative Pre‐to‐Post changes

**FIGURE 2:**
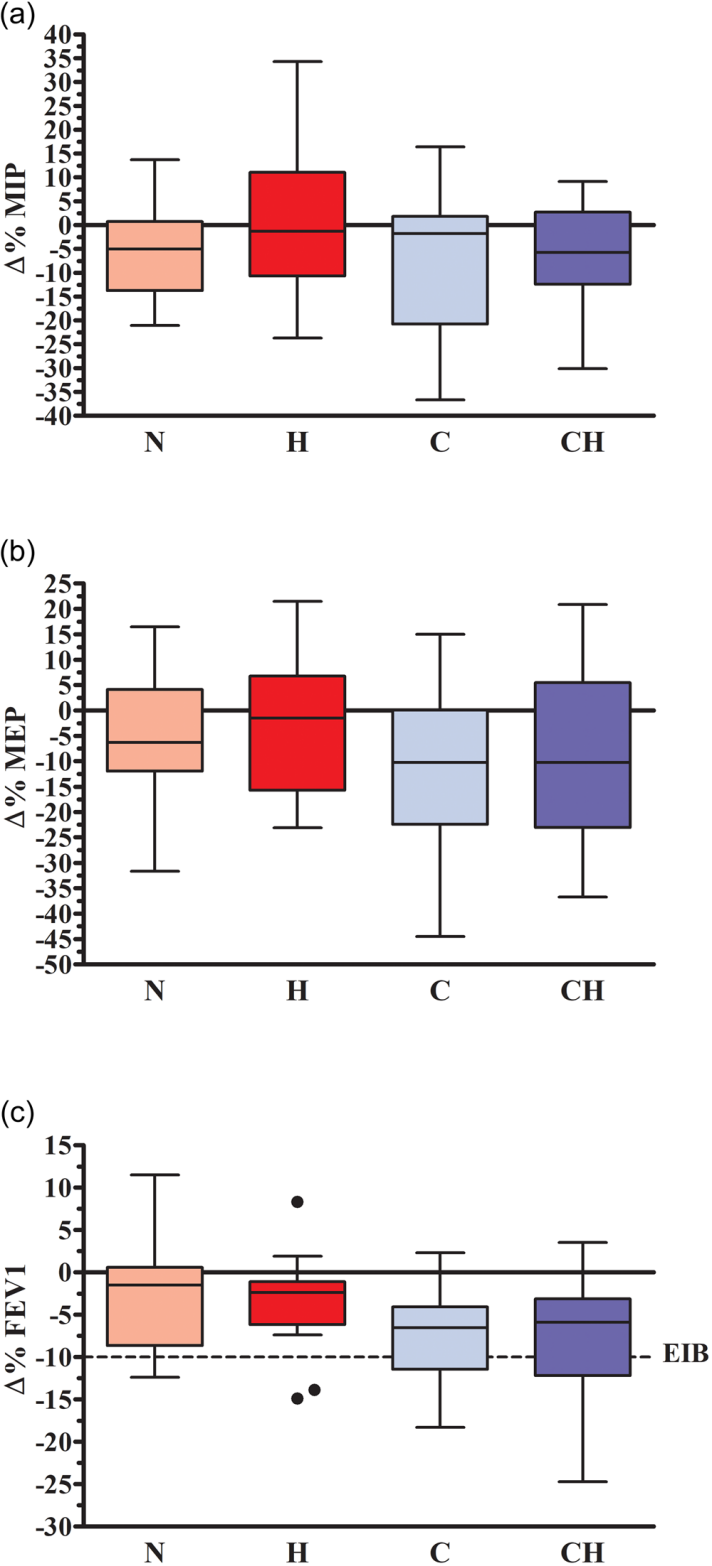
Percentage changes (∆%) from pretrial values in acute MIP (maximal inspiratory pressure, a), MEP (maximal expiratory pressure, b) and FEV1 (c) measured post‐exercise. Negative values show a decrease from pretrial values. The box represents the interquartile range (IQR) from the first to the third quartile; the whiskers extend to the minimum and maximum values within 1.5 times the IQR, and outliers are indicated as individual points beyond the whiskers. The dashed horizontal line represents the clinical cutoff for EIB presence detection. N: 18°C, 20.9% $F_{IO_{2}}$ (pink); H: 18°C, 13.5% $F_{IO_{2}}$ (red); C: −20°C, 20.9% $F_{IO_{2}}$ (light‐blue); CH: −20°C, 13.5% $F_{IO_{2}}$ (blue).

The outcomes for maximal Pre‐to‐Post changes in LF and RMS are shown in Table 2. Changes in MIP and MEP values showed no general effect of ‘$F_{IO_{2}}$ ’ or ‘temperature’, nor a ‘$F_{IO_{2}}$ × temperature’ interaction (see Figure 2a, b).

**TABLE 2:** Maximal decrease for MIP and MEP and FVC, FEV1, FEV1/FVC, PEF, FEF_25–75%_ and MEF_50%_ measurements post‐CPET in each environmental condition.

	N	H	C	CH	$F_{IO_{2}}$	Temp	$F_{IO_{2}}$ × temp
MEP (%)	−6.5 ±13.3 (14)	−2.7 ± 13.7 (14)	−12.3 ± 17.3 (14)	−7.9 ± 17.7 (13)	0.188	0.168	0.928
MIP (%)	−5.3 ± 10.3 (14)	1.8 ± 17.6 (14)	−7.5 ± 15.5 (13)	−6.4 ± 12.5 (13)	0.201	0.171	0.382
FVC (%)	−7.2 ± 6.8 (14)	−7.6 ± 7.3 (13)	−6.1 ± 4.8 (14)	−8.0 ± 5.2 (13)	0.284	0.619	0.423
FEV1 (%)	−2.8 ± 6.3 (14)	−3.7 ± 6.1 (13)	−7.6 ± 5.8 (14)	−7.6 ± 7.2 (13)	0.667	**<0.001**	0.589
FEV1/FVC (%)	1.8 ± 2.6 (14)	1.2 ± 3.7 (13)	−3.3 ± 3.5 (14)	−1.5 ± 4.4 (13)	0.468	**<0.001**	0.135
PEF (%)	−5.9 ± 9.3 (14)	−4.9 ± 6.2 (13)	−9.5 ± 6.9 (14)	−12.6 ± 10.5 (13)	0.571	**<0.001**	0.243
FEF_25–75%_ (%)	3.0 ± 10.8 (14)	0.3 ± 9.3 (14)	−11.6 ± 10.0 (14)	−6.8 ± 14.1 (13)	0.682	**<0.001**	0.197
MEF_50%_ (%)	1.5 ± 11.8 (14)	−3.0 ± 7.6 (13)	−10.4 ± 9.8 (14)	−12.0 ± 17.1 (13)	0.273	**<0.001**	0.532

Data reported as means ± SD (n). Delta changes are expressed as percentage change from pre‐test values. Bold characters represent statistical significance (P < 0.05). n: number of observations per experimental condition. Abbreviations: FVC, forced vital capacity; FEF25–75%, forced expiratory flow at 25–75%; FEV1, forced expiratory volume in 1 s; $F_{IO_{2}}$, fraction of Inspired oxygen; MEF50%, mid expiratory flow at 50%; MEP, maximal expiratory pressure; MIP, maximal inspiratory pressure; PEF, peak expiratory flow; temp, ambient temperature. N: 18°C, 20.9% $F_{IO_{2}}$; H: 18°C, 13.5% $F_{IO_{2}}$; C: −20°C, 20.9% $F_{IO_{2}}$; CH: −20°C, 13.5% $F_{IO_{2}}$.

Furthermore, no general effect of ‘$F_{IO_{2}}$ ’ nor ‘$F_{IO_{2}}$ × temperature’ interaction was found in maximum relative Pre‐to‐Post exercise changes for any of the considered LF variables. However, except for FVC, a general effect of ‘temperature’ was found in all other parameters (all *P <* 0.001), whose relative decrease from Pre‐exercise trial values was significantly higher in the cold conditions if compared to the normothermic ones. Figure 2c shows Pre‐to‐Post exercise changes in FEV1 in the four conditions. EIB prevalence considered as a ∆% change in FEV1 > 10% (Anderson & Daviskas, 2000) is presented in Table 3.

**TABLE 3:** Absolute Pre and Post exercise FEV1 measurements, as well as ∆% Pre‐to‐Post change in FEV1, for each tested subject.

	N			H			C			CH		
Subject	Pre	Post	∆%	Pre	Post	∆%	Pre	Post	∆%	Pre	Post	∆%
1	4.71	4.73	0.4	4.7	4.79	1.9	5.01	4.76	−5.0	4.72	4.57	−3.2
2	4.77	4.71	−1.3	4.7	4.5	−4.3	4.86	4.38	−**9.9**	4.26	4.06	−4.7
3	3.94	3.69	−6.3	4.05	3.98	−1.7	3.88	3.64	−6.2	4.06	3.71	−8.6
4	3.91	3.92	0.3	4.18	3.97	−5.0	4.04	3.76	−6.9	4.2	3.6	−**14.3**
5	4.13	4.06	−1.7	4.5	4.29	−4.7	4.53	4.26	−6.0	4.35	4.01	−7.8
6	4.2	3.68	−**12.4**	4.06	3.76	−7.4	4.2	3.43	−**18.3**	4.25	3.7	−**12.9**
7	3.05	3.4	11.5	3.14	3.4	8.3	3.22	3.18	−1.2	3.14	3.25	3.5
8	4.26	3.82	−**10.3**	4.04	3.44	−**14.9**	4.22	3.74	−**11.4**	3.96	2.98	−**24.7**
9	3.92	3.92	0.0				4.21	3.72	−**11.6**	3.66	3.66	0.0
10	4.43	4.48	1.1	4.57	4.5	−1.5	4.42	4.52	2.3	4.24	4.11	−3.1
11	4.58	4.45	−2.8	4.22	4.19	−0.7	4.51	4.17	−7.5	4.44	4.18	−5.9
12	4.92	4.52	−8.1	4.91	4.79	−2.4	4.95	4.71	−4.8	4.92	4.66	−5.3
13	4.59	4.67	1.7	4.5	4.4	−2.2	4.77	4.68	−1.9			
14	4.91	4.39	−**10.6**	4.74	4.08	−**13.9**	4.73	3.89	−**17.8**	4.86	4.3	−**11.5**
mean	4.31	4.17	−2.8	4.33	4.16	−3.7	4.40	4.06	−7.6	4.24	3.91	−7.6
SD	0.51	0.44	6.3	0.46	0.45	6.1	0.49	0.51	5.8	0.48	0.49	7.2
EIB			21%			14%			36%			29%

Numbers in bold indicate exercise‐induced bronchoconstriction (EIB) development. N: 18°C, 20.9% $F_{IO_{2}}$; H: 18°C, 13.5% $F_{IO_{2}}$; C: −20°C, 20.9% $F_{IO_{2}}$; CH: ‐20°C, 13.5% $F_{IO_{2}}$.

### 3.4 Relative lung function changes at each post‐exercise time point

**FIGURE 3:**
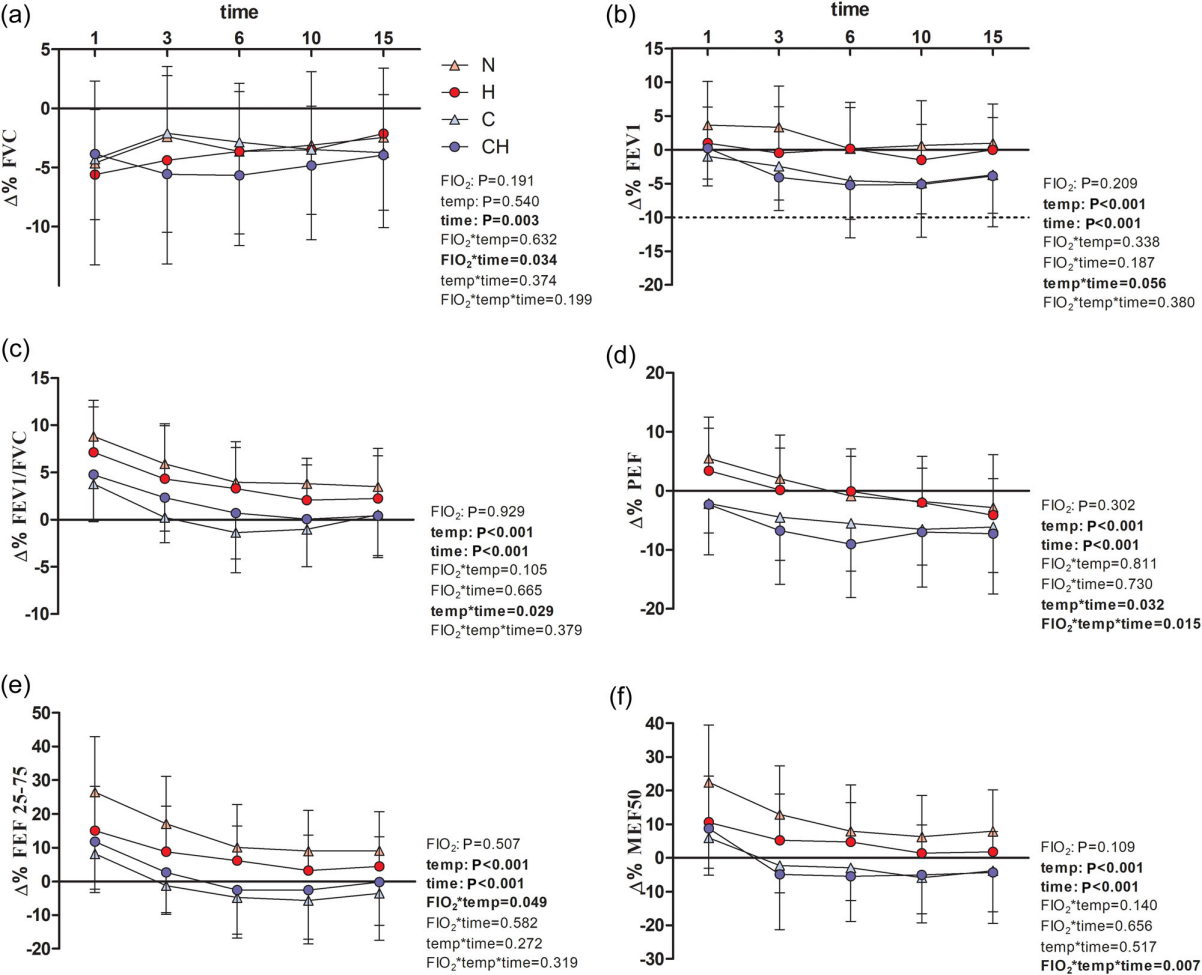
Percentage changes (∆%) from pretrial values in acute lung function recovery variables measured at each post‐exercise time point (1‐Post, 3‐Post, 6‐Post, 10‐Post, 15‐Post) for FVC (a), FEV1 (b), FEV1/FVC (c), PEF (d), FEF_25–75%_ (e) and MEF_50%_ (f). Normothermic normoxia (N, pink triangles), normothermic hypoxia (H, $F_{IO_{2}}$ 13.5%; red circles), cold normoxia (C,−20°C; light‐blue triangles) and cold‐hypoxia (CH, blue circles). Negative values show a decrease from pretrial values. Values are presented as means ± SD. FEF_25–75%_, forced expiratory flow at 25–75%; FEV1, forced expiratory volume in 1 s; FVC, forced vital capacity; PEF, peak expiratory flow; MEF_50%_, mid expiratory flow at 50%.

A graphical representation of the results is presented in Figure 3, displaying the main effects of hypoxia ($F_{IO_{2}}$), temperature (temp) and time (time), followed by two‐ and three‐way interaction effects. Considering FVC, no differences in single time point values between environmental conditions were seen, but a ‘$F_{IO_{2}}$ × time’ interaction revealed that FVC relative decrease was significantly higher at 3‐Post if compared to 15‐Post only in the hypoxic conditions, regardless of temperature. FEV1 changes were higher in the cold trials (i.e. general effect of ‘temperature’), in which this parameter decreased from 3‐Post to 10‐Post, starting to go back to normal values at 15‐Post (in the cold: 1‐Post > 3‐Post; 1‐Post > 6‐Post; 1‐Post > 10‐Post). A similar time‐trend was seen for FEV1/FVC%, despite this parameter showing higher values at 1‐Post in all conditions (if compared to pre‐trial values) and never falling significantly below pre‐exercising values. The general effect of cold was maintained also for PEF, as well as the ‘time’ effect, showing a decrease in this parameter from 1‐Post to 15‐Post in all conditions. However, a ‘$F_{IO_{2}}$ × temperature × time’ interaction effect showed that ∆% change in PEF shows a tendency to be higher in CH than in C alone at 3‐Post exercise (*P =* 0.069). Finally, also FEF_25–75%_ and MEF_50%_ showed similar general effects of ‘temperature’ and ‘time’, with both parameters increasing immediately post‐exercise cessation in all conditions (i.e. 1‐Post), and returning to baseline values in normothermia or below these values in the cold at 15‐Post.

### 3.5 Relationship between changes in LF, RMS and ventilatory data during exercise

**FIGURE 4:**
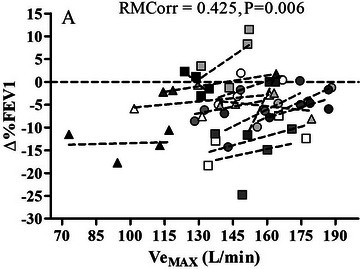
Relationship between Pre‐to‐Post exercise changes (∆%) in forced expiratory volume over 1 s (FEV1%) and ventilation at the end of CPET (${\dot{V}}_{\mathrm{Emax}}$). The same symbols represent the 4 experimental conditions for each subject; dashed lines represent single subject correlation considering the 4 experimental trials.

The within‐individual association between maximum Pre‐to‐Post exercise changes in MIP, MEP, FVC, FEV1, FEV1/FVC, PEF, FEF_25–75%_ and MEF_50%_ and maximal ventilatory data at the end of CPET (${\dot{V}}_{\mathrm{Emax}}$, *V*_tmax_, *f*_Rmax_) is presented in Table 4. Note that when dealing with data presented as ∆% changes from pretrial values, negative outcomes may be displayed (i.e. −10% of Pre‐test value); consequently, a positive correlation indicates that the greater is the decrease in the parameter, the lower is the value of absolute ventilatory responses during exercise (Figure 4, showing the relationship between ∆% change in FEV1 and ${\dot{V}}_{\mathrm{Emax}}$, is explicative of this concept).

**TABLE 4:** Results of repeated measures correlation analysis between MIP, MEP, FVC, FEV1, FEV1/FVC, PEF, FEF_25–75%_ and MEF_50%_ expressed as post‐exercise percentage changes (∆%)  from pretrial values and ventilatory parameters at the end of CPET (${\dot{V}}_{\mathrm{Emax}}$, *V*_tmax_, *f*_Rmax_).

		${\dot{V}}_{\mathrm{Emax}}$	*f*_Rmax_	*V*_tmax_
∆% MEP	r_rm_	0.259	0.035	0.224
	*P*	0.098	0.825	0.154
∆% MIP	r_rm_	0.219	0.074	0.095
	*P*	0.170	0.645	0.556
∆% FVC	r_rm_	−0072	**−0.359**	0.186
	*P*	0.653	**0.021**	0.243
∆% FEV1	r_rm_	**0.425**	−0.081	**0.436**
	*P*	**0.006**	0.613	**0.004**
∆% FEV1/FVC	r_rm_	**0.447**	0.194	0.263
	*P*	**0.003**	0.225	0.097
∆% PEF	r_rm_	0.239	−0.142	**0.355**
	*P*	0.133	0.376	**0.023**
∆% FEF_25–75%_	r_rm_	**0.329**	0.076	0.236
	*P*	**0.033**	0.630	0.133
∆% MEF_50%_	r_rm_	**0.497**	0.188	**0.308**
	*P*	**0.001**	0.240	**0.050**

Significant correlations have are presented in bold. Statistical significance was set at *P <* 0.05. Abbreviations: FEF_25–75%_, forced expiratory flow at 25–75%; FEV1, forced expiratory volume in 1 s; *f*_Rmax_, respiratory frequency at the end of maximal CPET; FVC, forced vital capacity; MEF_50%_, mid expiratory flow at 50%; MEP, maximal expiratory pressure; MIP, maximal inspiratory pressure; PEF, peak expiratory flow; r_rm_, repeated measures correlation; ${\dot{V}}_{\mathrm{Emax}}$, ventilation at the end of maximal CPET; *V*_tmax_, tidal volume at the end of maximal CPET.

## 4 DISCUSSION

Despite real‐life high altitude exposure often occurring concurrently with extremely cold temperatures, studies on their combined effect on several physiological mechanisms that may limit exercise practice and/or pose health risks for practitioners are highly under‐represented in literature (Mugele et al., 2021; Tipton, 2012). To the best of our knowledge, this is the first study evaluating together Pre‐to‐Post exercise variations in LF and RMS in a cold‐hypoxic environment, as well as their relationship to ventilatory responses at peak exercise. The key findings of this study confirmed previously demonstrated cold‐induced impairment of LF following high‐intensity exercise (Kennedy & Faulhaber, 2018; Kennedy et al., 2019), also showing a relationship between this phenomenon and peak ventilatory responses, which were the lowest in the cold, indicating the possible presence of airflow limitations already during exercise in this condition. On the other hand, consistently with no changes in RMS from Pre‐to‐Post exercise under any environmental condition, no effect of hypoxia on maximal ventilatory responses directly attributable to decreased Post‐exercise RMS in this condition was observed.

Consequently, cold‐hypoxic exposure did not superimpose RMF and LF impairments following a discontinuous maximal test to exhaustion, but the ventilatory pattern adopted in CH suggests a possible interactive effect of the two conditions on respiratory system responses to exercise, which needs further investigation.

The absence of significant Pre‐to‐Post changes in RMS was unexpected, especially under hypoxic conditions, as Oueslati et al. (2018) demonstrated a ∼13% significant decrease in both MIP and MEP values after maximal incremental running and cycling tests to exhaustion already in normoxic conditions. Moreover, Hinde et al. (2018) found an additive effect of cold and hypoxia ($F_{IO_{2}}$ 11.8%, temperature −10°C) on MIP decrements after an approximately 2‐h walk at different speeds and slopes while maintaining an exercising intensity of ∼40% ${\dot{V}}_{O_{2}\max}$. It is possible that the intermittent nature of the incremental test in the presented study (4 min of work interspersed by a 2‐min recovery period) allowed the respiratory muscles to recover, potentially delaying the onset of fatigue (Kurti et al., 2015). Additionally, measuring RMS as an indirect index of fatigue may have contributed to the subdued results. In fact, Gudjonsdottir et al. (2001) found that diaphragmatic force‐generating capacity, assessed through transdiaphragmatic pressure changes, was significantly reduced after a 10‐min incremental test to exhaustion at high altitude compared to equivalent work rates at sea level, concluding that hypoxia alone was the primary factor behind these impairments. MIP and MEP assessments provide an indirect measure of overall RMF but do not distinguish between the specific contributions of the diaphragm and accessory respiratory muscles, which may result in biased outcomes.

Concerning LF responses to cold environments, research has yielded heterogeneous findings. Kennedy and Faulhaber (2018), Kennedy et al. (2020, 2019) reported mean decreases of 4–7% in FEV1 and 10–12% in FEF_25–75%_, FEF_50%_ and PEF following short‐duration high‐intensity exercise (i.e. 8–20 min in the severe domain) when exposed to ambient temperatures between −15 and −20°C. In these studies, comparable responses were found between trained men and women (Kennedy et al., 2020) and between subjects of different training levels (${\dot{V}}_{O_{2}\max}$ between ∼41 and 70 mL/kg/min) (Kennedy & Faulhaber, 2018). Similarly, Therminarias et al. (1989) documented a 5% decline in FEV1 and a 10% reduction in FEF_75%_ after maximal cycling at −10°C in well‐trained cyclists.

In contrast, Eklund et al. (2022) observed only an ∼1.7% decrease in FEV1 after two 15‐min running bouts at −15°C, either at heavy (Eklund et al., 2022) or at moderate (Eklund et al., 2021) exercise intensity (i.e., 85% and 70% of ${\dot{V}}_{O_{2}\max}$, respectively). Interestingly, the latter study showed no peripheral bronchodilatation in the cold, whereas significant increases were observed after exercise at +10°C (i.e. increased reactance compared to baseline values). This finding suggests that despite no major differences in FEV1, low ambient temperatures still elicit distinct lung function responses compared to normothermic conditions. Additionally, Carey (2015) and Helenius et al. (2002) reported minimal alterations in FEV1 following short‐duration heavy‐intensity exercise at ambient temperatures between −10 and −5°C.

The conflicting outcomes appear to be primarily related to the severity of the exercise stimulus (rather than its duration) and the degree of cold exposure, with greater impairments observed at temperatures below −10°C. In contrast, subjects’ training status and relative ventilatory demands seem to play a lesser role. Importantly, all the aforementioned results pertain to subjects with no prior diagnosis of EIB, making them directly comparable to our sample. However, when considering individuals with diagnosed EIB, maximal FEV1 decreases of −24% have been reported after 8 min of intense exercise at +20°C, reaching −31% at −20°C (Stensrud et al., 2007). Future studies should precisely define these factors and avoid generalizing results across different temperatures, exercise intensities and populations.

From a clinical perspective, EIB is diagnosed when the Pre‐to‐Post exercise FEV1 decreases by at least 10% (Anderson & Daviskas, 2000). Based on this criterion, our results showed that EIB prevalence was highest in cold conditions, reaching 36% in C and 29% in CH (see Table 2). However, previous studies (Helenius et al., 2002) suggested that post‐exercise FEV1 reductions of 6% or more should already be considered abnormal in elite athletes undergoing outdoor exercise challenge tests, despite not being classified as clinically significant. Looking at individual data in Table 2, it is evident that cold conditions led to a higher frequency of abnormal lung function responses compared to normothermic trials. This highlights the need for a better understanding of pulmonary responses to extreme cold conditions and their potential impact on exercise capacity and overall health of practitioners.

Interestingly, our findings demonstrate that while small airway function increases immediately post‐exercise across all conditions (i.e. increased FEF_25–75%_ and MEF_50%_ at Post‐1), this effect is sustained for 15 min after exercise cessation only in normothermic trials. Eklund et al. (2022) identified peripheral bronchodilatation after heavy exercise at −15°C but not after time‐matched moderate‐intensity exercise at −10.7°C (Eklund et al., 2021). Additionally, Kennedy et al. (2020) reported increased expiratory flow rates after 8 min of severe‐intensity exercise at 0°C but not at −20°C. Furthermore, Gavrielatos et al. (2022) found no significant differences in FEV1 changes after 30 versus 90 min of moderate‐intensity exercise (60% ${\dot{V}}_{O_{2}\max}$) at −15°C in trained men and women, though atopic individuals exhibited increased post‐exercise reactance, indicating enhanced lung elasticity due to peripheral bronchodilatation. Collectively, these findings underscore the necessity of considering temperature thresholds and exercise intensity as key determinants of respiratory adaptations, emphasizing the importance of refined methodologies to elucidate pulmonary responses in extremely cold environments.

A novelty of this study resides in the results of the multiple correlation analysis, which revealed a trend towards lower ${\dot{V}}_{E}$ and *V*_t_ values during exercise (i.e. in the cold) being associated with worsened post‐exercise airway responses. Mediano et al. (2017) demonstrated high dynamic hyperinflation (DH) prevalence during exercise in patients with asthma and EIB if compared to patients with asthma alone and healthy controls. DH refers to the temporary increase in operating lung volumes above their resting values (i.e. increased end expiratory lung volume, EELV), reflecting compromised lung emptying and air trapping during tidal breathing (Stickland et al., 2022). Increased EELV may be the cause of decreased *V*_t_ (and consequently ${\dot{V}}_{E}$) at maximal exercising intensities in the cold found in this study, but measurements of inspiratory capacity during exercise in low ambient temperature are necessary to gain a more comprehensive understanding of this phenomenon, especially when considering non‐asthmatic subjects. Whether dynamic hyperinflation could be an early marker of EIB or it could contribute to its development is still a matter of debate. In this context, it may also be worth considering the role of heat exchanger masks, which are designed to reduce airway cooling and drying and may mitigate bronchoconstriction, although they might slightly increase the work of breathing and limit heat dissipation (Hanstock et al., 2020). Investigating how such devices interact with ventilatory patterns and dynamic hyperinflation in cold environments could provide further insight into the physiological mechanisms underpinning EIB.

Regarding cold‐hypoxic exposure, the decrease in ${\dot{V}}_{\mathrm{Emax}}$ exhibits relative rather than complete additive effects (H: −6.9%, C: −14.6%, CH: −16.6%), whereas *V*_tmax_ shows a complete additive reduction (H: −7.05%, C: −9.57%, CH: −18.65%), requiring further investigation. While ventilation in hypoxia increases at submaximal intensities, it remains similar to or even lower than in normoxia at maximal exercise intensity, primarily due to the associated decline in exercise capacity (Callovini et al., 2023). However, once exposed to CH, it seems that the worst‐strain‐takes‐precedence principle took place (Lloyd et al., 2016), avoiding ${\dot{V}}_{\mathrm{Emax}}$ disproportionately decreasing in this condition through an increase in *f*_Rmax_ if compared to single stressor exposure (CH vs. H: + 5.7% and CH vs. C: + 7.0%). This was possible because the proposed study design did not actually superimpose RMF on LF impairments during combined stressors exposure, allowing the respiratory muscles to sustain an increased *f*_R_ to overcome the further decrease in *V*_t_. However, shallow and rapid breathing increases the movement of air in the anatomical dead space, whereas slower, deeper breaths are more effective at delivering air to the gas exchange sites in the lungs (Richard & Koehle, 2012); this means that despite small differences in ${\dot{V}}_{\mathrm{Emax}}$ between C and CH, the ventilatory pattern adopted in the latter condition may decrease effective ventilation for gas exchange occurrence, being one of the possible explanations for complete additive effect of C and H on WL_max_ decrease in CH (Callovini et al., 2023). Moreover, these changes in the ventilatory strategy adopted during exercise in CH may cause the diaphragm to work at a suboptimal portion of the length–tension curve, favouring RMF if longer exercising tasks are considered (Mediano et al., 2017; Price, 2014).

The limitations of this work certainly pave the way for future studies. Firstly, the exercise modality adopted (i.e. a submaximal‐to‐maximal intermittent test to exhaustion) may have attenuated the hypoxic effect on respiratory muscle strength (RMS). Future investigations should consider protocols involving longer exercise durations at a sustained high intensity (e.g., 2 × 15 min at 85% ${\dot{V}}_{O_{2}\max}$ with 5 min of recovery between bouts, as proposed by Eklund et al., 2022) to evaluate whether the simultaneous occurrence of EIB and RMF can be observed in the cold‐hypoxic trial. However, our outcomes confirm that to mitigate the impact of exercising under extreme environmental conditions, it may be beneficial to implement shorter exercise periods along with frequent breaks during hikes at high altitudes (Fornasiero et al., 2020).

Moreover, normobaric hypoxia, as opposed to hypobaric hypoxia, subjected participants to possibly greater flow limitations due to the unchanged air density, which does not mimic the conditions of actual high‐altitude exposure and may have further influenced the results (Cogo et al., 1997; Deboeck et al., 2005). Thus, a comprehensive consideration of all these aspects when exposed to hypobaric hypoxic conditions should be carried out. Finally, the inclusion of only male participants in this study limits the generalizability of the findings to females. This design choice aimed to minimize variability arising from sex‐based physiological differences in respiratory responses to exercise under extreme environmental conditions (Raberin et al., 2023). Future research should incorporate female participants to assess whether similar responses occur across sexes and to improve the broader applicability of these findings.

In conclusion, our findings reveal that post‐exercise lung function is negatively impacted by cold exposure, whereas the effects of hypoxia on the respiratory system, both as an independent stressor and in combination with cold, were not apparent. This issue seems, however, to require further investigation, specifically when considering longer exercise durations spent at or above 85% of ${\dot{V}}_{O_{2}\max}$. Furthermore, a relationship between ventilatory responses to exercise and LF impairments has been found, suggesting that the development of EIB post‐exercise cessation and the manifestation of mechanical constraints to ventilation during exercise may be, to some extent, related. A deeper understanding of these factors is essential for minimizing the risks and exercising safely in extreme environmental conditions.

## AUTHOR CONTRIBUTIONS

Callovini, A. Fornasiero, A. Savoldelli, G. Dorelli, M. Decet, L. Bortolan, B. Pellegrini and F. Schena participated in study conception and design. A. Callovini, A. Fornasiero and M. Decet participated in data acquisition. A. Callovini, A. Fornasiero, M. Decet and L. Bortolan participated in data analysis. A. Callovini. A. Fornasiero and G. Dorelli were responsible for data interpretation. A.Callovini contributed to the draft of the paper. All authors critically reviewed and approved the final version of the manuscript. All authors agree to be accountable for all aspects of the work in ensuring that questions related to the accuracy or integrity of any part of the work are appropriately investigated and resolved. All persons designated as authors qualify for authorship, and all those who qualify for authorship are listed

## CONFLICT OF INTEREST

None declared.

## Supporting information



Table S1. Absolute Pre and Post incremental test MIP, MEP, FVC, FEV1, FEV1/FVC, PEF, FEF_25–75%_ and MEF_50%_ measurements in each environmental condition.

## Data Availability

The data that support the findings of this study are available from the corresponding author upon reasonable request.
