# Supplementary material for: Combined effects of normobaric hypoxia and cold on respiratory system responses to high‐intensity exercise
Source: Exp Physiol. 2025 May 11;110(12):1892–903. doi: 10.1113/EP092468 (PMC12665948; doi:10.1113/EP092468)
Supplement: Supplementary file 1 — Table S1. Absolute Pre and Post incremental test MIP, MEP, FVC, FEV1, FEV1/FVC, PEF, FEF25–75% and MEF50% measurements in each environmental condition. [file EPH-110-1892-s001.DOCX]

**SUPPLEMMENTAL MATERIAL**

Table S1 Absolute PRE and POST incremental test MIP, MEP, FVC, FEV1, FEV1/FVC, PEF, FEF25-75 and MEF 50 measurements in each environmental condition.

| **Characteristics** | | **N** | | | | | | | | **H** | | | | | | | | **C** | | | | | | | | **CH** | | | | | | | |
| --- | --- | --- | --- | --- | --- | --- | --- | --- | --- | --- | --- | --- | --- | --- | --- | --- | --- | --- | --- | --- | --- | --- | --- | --- | --- | --- | --- | --- | --- | --- | --- | --- | --- |
|  |  | **pre** | | |  | **post** | | |  | **pre** | | |  | **post** | | |  | **pre** | | |  | **post** | | |  | **pre** | | |  | **post** | | |  |
|  |  | **mean** | **±** | **SD** | **(n)** | **mean** | **±** | **SD** | **(n)** | **mean** | **±** | **SD** | **(n)** | **mean** | **±** | **SD** | **(n)** | **mean** | **±** | **SD** | **(n)** | **mean** | **±** | **SD** | **(n)** | **mean** | **±** | **SD** | **(n)** | **mean** | **±** | **SD** | **(n)** |
| **MEP** | cmH2O | 144.0 | **±** | 50.4 | [14] | 137.0 | **±** | 58.5 | [14] | 137.6 | **±** | 42.9 | [14] | 135.4 | **±** | 53.0 | [14] | 144.8 | **±** | 51.0 | [14] | 124.2 | **±** | 41.1 | [14] | 141.3 | **±** | 49.8 | [13] | 133.6 | **±** | 60.2 | [13] |
| **MIP** | cmH2O | 119.8 | **±** | 26.1 | [14] | 113.9 | **±** | 30.7 | [14] | 115.0 | **±** | 25.6 | [14] | 117.1 | **±** | 32.9 | [14] | 129.9 | **±** | 32.9 | [13] | 117.7 | **±** | 36.6 | [14] | 119.9 | **±** | 27.7 | [13] | 112.9 | **±** | 32.7 | [13] |
| **FVC** | L | 5.5 | **±** | 0.6 | [14] | 5.1 | **±** | 0.6 | [14] | 5.5 | **±** | 0.6 | [13] | 5.1 | **±** | 0.7 | [13] | 5.6 | **±** | 0.6 | [14] | 5.2 | **±** | 0.6 | [14] | 5.4 | **±** | 0.5 | [13] | 5.0 | **±** | 0.5 | [13] |
| **FEV1** | L | 4.3 | **±** | 0.5 | [14] | 4.2 | **±** | 0.4 | [14] | 4.3 | **±** | 0.5 | [13] | 4.2 | **±** | 0.5 | [13] | 4.4 | **±** | 0.5 | [14] | 4.1 | **±** | 0.5 | [14] | 4.2 | **±** | 0.5 | [13] | 3.9 | **±** | 0.5 | [13] |
| **FEV1/FVC** | Ratio | 78.3 | **±** | 5.1 | [14] | 79.7 | **±** | 5.8 | [14] | 79.0 | **±** | 5.0 | [13] | 79.9 | **±** | 5.5 | [13] | 79.0 | **±** | 6.0 | [14] | 76.4 | **±** | 5.8 | [14] | 78.0 | **±** | 6.5 | [13] | 76.9 | **±** | 7.9 | [13] |
| **PEF** | L/sec | 10.4 | **±** | 1.5 | [14] | 9.8 | **±** | 2.0 | [14] | 10.4 | **±** | 1.4 | [13] | 9.9 | **±** | 1.7 | [13] | 10.6 | **±** | 1.4 | [14] | 9.7 | **±** | 1.7 | [14] | 10.2 | **±** | 1.3 | [13] | 9.0 | **±** | 1.6 | [13] |
| **FEF25-75** | L/sec | 3.9 | **±** | 0.9 | [14] | 4.0 | **±** | 1.0 | [14] | 4.0 | **±** | 0.8 | [13] | 4.0 | **±** | 0.8 | [13] | 4.1 | **±** | 1.0 | [14] | 3.6 | **±** | 0.9 | [14] | 3.8 | **±** | 0.9 | [13] | 3.5 | **±** | 1.0 | [13] |
| **MEF50** | L/sec | 4.5 | **±** | 1.0 | [14] | 4.5 | **±** | 1.1 | [14] | 4.6 | **±** | 0.9 | [13] | 4.5 | **±** | 0.9 | [13] | 4.6 | **±** | 1.0 | [14] | 4.1 | **±** | 1.0 | [14] | 4.4 | **±** | 1.1 | [13] | 3.8 | **±** | 1.2 | [13] |

Data reported as mean ± SD. MIP, maximal inspiratory pressure, MEP, maximal expiratory pressure; FVC, forced vital capacity; FEV1, forced expiratory volume in 1 s; PEF, peak expiratory flow; FEF 25--75, forced expiratory flow at 25%–75%, MEF 50, mid expiratory flow at 50%. N: 18°C, 20.9% FIO_2;_ H; 18°C, 13.5% FIO_2;_ C: -20°C, 20.9% FIO_2;_ CH: -20°C, 13.5% FIO_2_ . n: number of observations per experimental condition.
